# Secondary Bioactive Metabolites from Plant-Derived Food Byproducts through Ecopharmacognostic Approaches: A Bound Phenolic Case Study

**DOI:** 10.3390/plants9091060

**Published:** 2020-08-19

**Authors:** Ilaria Burlini, Gianni Sacchetti

**Affiliations:** Department of Life Sciences and Biotechnology—Pharmaceutical Biology Lab., Research Unit 7 of the Terra & Acqua Tech Technopole Lab., University of Ferrara, P.le Chiappini 2, 44123 Ferrara, Italy; ilaria.burlini@unife.it

**Keywords:** food waste, byproducts, green extractions, bound phenolics

## Abstract

The climate emergency and the risks to biodiversity that the planet is facing nowadays, have made the management of food resources increasingly complex but potentially interesting. According to FAO, one-third of the edible parts of food produced throughout the whole food supply chain gets lost or wasted globally every year. At the same time, demographic growth makes it necessary to change course toward sustainable economic development in order to satisfy market demands. The European Union supported the idea of a Circular Economy from 2015 and arranged annual Action Plans toward a greener, climate-neutral economy. Following the biorefinery concept, food waste becomes byproducts that can be recovered and exploited as high added-value materials for industrial applications. The use of sustainable extraction processes to manage food byproducts is a task that research has to support through the development of low environmental impact strategies. This review, therefore, aims to take stock of the possibilities of extracting molecules from food waste biomass following ecopharmacognostic approaches inspired by green chemistry guidelines. In particular, the use of innovative hybrid techniques to maximize yields and minimize the environmental impact of processes is reviewed, with a focus on bound phenolic extractions.

## 1. Introduction and Historical Background

Food waste recovery has become a hot topic in the field of natural product and ecopharmacognosy research in the past 20 years: however, it should not be surprising that the development of this research topic was born in conjunction with a series of ideologies and historical evidence. From the early roots of Carl Linnaeus, Alexander von Humboldt, Alfred Russel Wallace, Charles Darwin, Ernst Haeckel and many more contributors, ecology has been recognized as a science and it started to spread all over the scientific world as the study of all those manifold interactions between organisms and their environment. From the first half of the 20th century, ecology has reshaped the position of the human being that has become a crucial part of the biological equilibrium of the planet, now conceived as a finite space: the idea that the planetary biomass is limited makes the life—with its fragile balances among multiple and complex interactions—incapable of eternal renewal and subjected to dangerous imbalances for the survival of all the species on Earth, mankind included [1]. In 1981, Walter R. Stahel began developing the idea of sustainability by supporting “service—life extension of goods—reuse, repair, remanufacture, upgrade technologically” or what is known today as a circular economy [2]. The purpose of a circular economy, now supported by the European Commission action plans from 2015, is shifting from the linear scheme “taking, making, consuming, throwing away” to a circular flow which maintains goods and resources in the production economy as far as possible, reducing environmental impacts while maximizing resource efficiency promoting what is now known as the “cradle to cradle” way of production for the supply chains [3]. The birth of Industrial Ecology, announced by Erkman in 1997, represents another useful tool for understanding the circular flows of materials and energy and, therefore, the creation of alternative managements through reuse, repair, recycling and remanufacturing for the recovery of components and extend their lifecycle [4]. The recovery of industrial byproducts and the study of their potential applications began already before the First World War, accentuated in the first postwar period with the increase in the prices of basic necessities and developing further from the middle of the last century [5]. However, a significant interest increase by academic research coincided, between the 1960s and 1970s, with a political awareness of the importance of minimizing waste (in that period Mobius Loop was introduced as a figurative expression of Reducing, Reusing, Recycling), thus a large number of publications about sustainable strategies of waste valorization enriched the literature [6]. The recent updates given by the world’s leading climate science during the UN Intergovernmental Panel on Climate Change (IPCC) held at the end of 2018 regarding the environmental risks of the planet, pointed out that urgent and unprecedented changes are needed to avoid catastrophic environmental breakdown. The management of the planet’s resources and wastes is, consequently, stimulating research to find useful and alternative strategies. 

This review is focused on ecopharmacognostic approaches as opportunities to valorize food wastes/byproducts as sources of healthy and useful bioactives, giving a general overview of food wastes/byproducts general definitions, costs and current management, as well as promising examples of alternatives to petroleum-derived solvent extraction techniques. Conventional extraction processes are in many respects no longer sustainable in terms of energy consumption, environmental impact, solvent biodegradability and thus not effective in yields and selectivity [7]. The ecopharmacognostic approach to obtain bioactive molecules for applicative purposes—health included—is the way to go for a more sustainable production policy, respecting plant biodiversity through the best exploitation of plant resources.

## 2. Food Losses and Wastes

### 2.1. Definitions

The issues of food losses and wastes are of high importance as they are directly linked to the environment, economic development, food quality and safety and to food sustainability for developing countries. Hence, before going into the topic in detail, few essential definitions are given below. According to Fusions’s “Estimates of European food wastes levels”, “Food” is defined as “any substance or product, whether processed, partially processed or unprocessed, intended to be, or reasonably expected to be, eaten by humans. Food includes drink, chewing gum and any substance, including water, intentionally incorporated into food during its manufacture, preparation or treatment”. In the same way, food wastes are considered as “fractions of food and inedible parts of food removed from the food supply chain to be recovered or disposed of through composted, crops plowed in/not harvested, anaerobic digestion, bioenergy production, cogeneration, incineration, disposal to sewer, landfill or discarded to sea” [8]. A difference in meaning between “food waste” and “food losses” is given by the Food and Agricultural Organization of the United Nations (FAO): the former is considered as food loss which occurs at the end of the food chain and is related to the behavior of retailers and consumers, whereas the latter takes place at production, postharvest and processing stages in the food supply chain [9].

### 2.2. Types of Food and Waste (or ByProducts) Management

There are five stages in the food life cycle where waste is generated from both animal and vegetable sources: (1) agricultural production, (2) postharvest handling and storage, (3) processing, (4) distribution and (5) consumption, considered and described in the following table.

**Figure 1:**
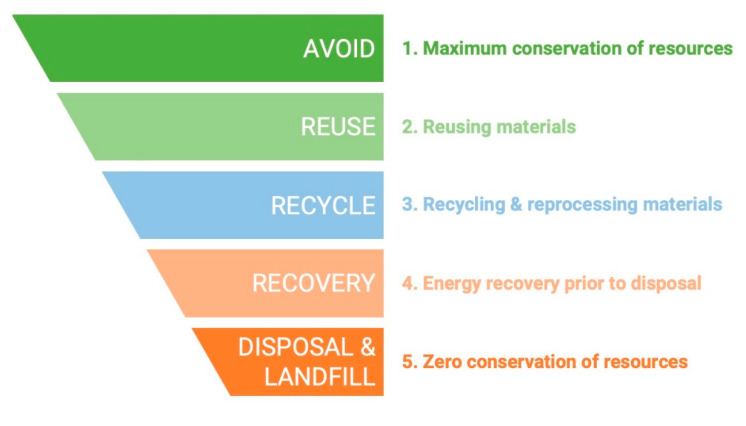
Hierarchy for waste processing.

Until a few decades ago, food wastes were considered neither a cost nor a benefit: they were used as animal feed, brought to landfills or sent for composting. The growing environmental issues as well as the high disposal costs are two of the main reasons that brought countries to a more sustainable approach. According to the Directive 2008/98/EC, adopted by the New Waste Framework Directive, the prevention of waste generation has to be a priority, followed by processing for reuse and recycling, with disposal and landfill as the least favored stages of waste management (Figure 1). Vision 2020, launched by ReFood in 2011, is a good example of a credible initiative aimed to ban food waste from landfill sites by 2020 in the UK [10,11,12].

Besides their pollution and hazardous aspects, in many cases food wastes might have a potential as recycled raw materials, converting to high-added-value products, or they could be raw materials for other industries. Particularly, the recovery of food residues is receiving increased attention because of the growing awareness of the benefits deriving from potentially marketable components present in food wastes which represent a possible and usable resource to obtain useful products [12,13].

Two generations of food waste valorization strategies are distinguished: the first-generation is aimed to use the complete material streams for animal feed, energy or compost production (e.g., bioenergy production); the second-generation valorization rely on recovery and conversion of specific components in order to obtain various classes of products (e.g., chemicals, bioactives, biofuels, etc.). Interesting sources of plant-derived food wastes can be found in each one of the previously described categories (Table 1), from agricultural wastes until those derived by consumption (municipal waste), thus much of the efforts in waste processing have been focused on this topic. During industrial processing a wide range of materials is generated and, apart from wastes that industry has to eliminate by disposal centers, incineration, or landfill, they can be distinguished in byproducts and coproducts. According to Chemat et al. [14], “byproducts” are unintentional and unpredictable residual products that appear during the extraction process. They can be used directly or as ingredients to manufacture another finished product, generating a new economic value. “Coproducts” are materials, intentional and unavoidable, produced along with the main product and with the same importance. Coproducts must always meet specifications for their characteristics and may be used directly for a particular application [14]. Recent studies focused on the exploitation of food waste show many possibilities for food supply chain waste recovery such as the valorization of wastes into high-value chemicals.

### 2.3. Extend, Costs and Global Concern

According to FAO, one-third of the edible parts of food produced for human consumption gets lost or wastes globally corresponding to 1.3 billion ton per year with a global cost of $750 billion annually [9]. As previously described, food wastes and losses are produced throughout the whole food supply chain but dramatic differences between industrialized countries and developing countries are reported: low-income countries counted more than 40% of food losses during the postharvest and processing levels while, differently, in medium and high-income countries more than 40% of food waste is produced at the consumer level (222 million tons). This means that food is thrown away even if it is still suitable for human consumption. It must make us reflect that this amount is almost as high as the total net food production in sub-Saharan Africa which counts 230 million tons [9]. FAO estimated that the societal costs of food wastage amounted to about $2.6 trillion in 2014: one trillion of them are costs from economic losses, $700 billion are societal costs of environmental impact and $900 billion are due to individual well-being losses [15].

Beside the mere economic loss, food wastes are responsible for dramatic Greenhouse Gases emissions (GHG) which, counting more than 8% of the total human GHG products, are almost as high as those derived by road transports. Climate changes and food wastes are closely related also in terms of water consumption, risks to biodiversity and soil erosions demonstrating the deep impact of this phenomenon on our society [16]. 

European amounts of food waste have been estimated for the year 2012 by Fusion’s project “Estimates of European food waste levels” for the European Commission and published in 2016 (project supported by the European Community’s Seventh Framework Programme under Agreement No. 311972) [8]. Thanks to a combination of national waste statistics and literature data collected within EU member States, it has been estimated the amount of food waste, including food and inedible parts associated with food. Resulting data confirmed the world estimations previously reported, evidencing household wastes as the most contributing sector to food waste (47 million tons), followed by processing (17 million tons). These numbers tell us that 72% of EU food waste comes from the aforementioned two sectors, while the remaining 28% is distributed among food service (11 million tons), production (nine million tons) and wholesale and retail (five million tons). World percentages of food losses and waste by production processes are reported in Table 2. In 2012, EU production of food wastes corresponded to 173 kg per capita (88 million tons whose costs amount approximately to 143 billion euros) and it has been estimated that these numbers could increase up to 30% by 2020 if no action is taken [8].

The Italian Parliament approved in 2016 a law against food waste (Law 166/2016 of 19 August 2016, n. 166) that has been considered the last step of the National Food Waste Prevention Plan [17] (Piano Nazionale di Prevenzione Degli Sprechi Alimentari (PINPAS)), with the aim of promoting the recovery and donation of food surpluses for charitable purposes and minimizing the negative impacts on the environment and on natural resources (reducing waste generation, encouraging reuse and recycle, extending products life). Italian food wastes have been counted around 5.1 million tons per year, and it has been expected that the new law will help to recover one million tons of food per year. This new Italian approach to food waste is just one step aimed to fight this evergreen and growing issue: to give an idea of the severity of the problem, one-quarter of the Italian forests serve just to absorb carbon dioxide emission produced as a result of the food waste production. Moreover, it has been calculated that if food waste was a Country, it would be the third-largest “emitter” of CO_2_ worldwide (just behind the USA and China) [18].

### 2.4. The EU Agenda against Food Wastes: The “Circular Economy Action Plan”

The most relevant opportunity to rebalance the food supply chain at the European level and develop a sustainable, low carbon, resource-efficient and competitive economy, is represented by the so-called “circular economy”. Shifting from “taking, making, consuming, throwing away” traditional linear scheme (“from cradle to grave”) to a circular model which closes the loop (“from cradle to cradle”), is the main purpose of this action plan. The Juncker Commission of the European Parliament released a proposal for the circular economy in 2015, aimed to amend the already cited 2008 Waste Framework Directive. By maintaining products, materials and resources in the economy as far as possible, the circular economy will boost the EU’s competitiveness in business, it will save energy and will reduce CO_2_ emissions. One key point stressed by the Commission to the European Parliament in 2015 refers to food waste as possible raw materials to be reused and injected back to the economy. For example, organic wastes could return back to the soil as sustainable fertilizers. Biomass and bio-based materials are other possible candidates as they can provide alternatives to fossil-based products and energy, and for this reason, the bio-based sector is supported by the EU with investments and projects through research funding. The Horizon 2020 work program 2016–2017 included the initiative “Industry 2020 in the circular economy” with funding for over €650 million for projects that supported the circular economy package [18]. In January 2017, a report on the implementation of the Circular Economy Action Plan was presented by the Commission to the EU Parliament confirming its full commitment. Strategies on plastic recycling and reuse, chemicals and wastes facilitation of management as well as dialogue with stakeholders are essential actions in order to make the circular economy a real growing and production system [19]. After intense debates among political groups, in March 2017 some relevant points have been prioritized: the need to give detailed definitions of “food waste”, the need to reduce food waste up to 30% by 2025 and up to 50% by 2050 compared to the 2014 baseline, as well as the need to create an efficient monitoring system by 2017 [16]. Another report on the implementation of the Circular Economy Action Plan was published in March 2019 and some relevant key points have been stressed, among them building circular models of production giving importance to Circular Design and Production Processes (Ecodesign Working Plan 2016–2019); empowering consumers who will be able to make conscious choices based on reliable information; turning wastes into sources as secondary raw materials (revised waste legislative framework); proposing a new regulation for secondary raw material recover (Fertilizing Products regulation); building up strategies for plastic lifecycle [20].

All these points together with the already described actions, will accelerate the transition toward a greener, and climate-neutral economy [20]. Finally, all these considerations and actions are reported in the so-called Agenda 2030 [21], where the ecopharmacognostic approaches to biomolecules production totally fit with its goals, and where “sustainability” and “circularity of processes” are the key words for sustainable development in many industrial and social fields.

## 3. Natural Products Research

### 3.1. The Role of Natural Products Research

In the presence of a realistic threat against natural resources and biodiversity, there is a need to find green solutions to tackle food waste and meet the growing demand for bio-based products—for e.g., bio-based cosmetics, nutraceuticals, health products, etc.—and natural products research has a crucial role in developing more and more sustainable strategies. Pharmacognosy, defined as “the study of biologically active natural products”, is a broad-based science with many applications from agricultural, to health and food industry. It is because of its multidisciplinary approach that it has become necessary to bring new theoretical and practical focuses on this science in order to direct scientists” practices in green terms. According to Cordell [22], “the challenge for researchers is how to best develop, innovate, and apply new strategies using knowledge in a sustainable manner, by considering and integrating the relevant cognate technologies and ideas”. Using knowledge in a sustainable manner means also giving intellectual property rights protection to indigenous groups who hold important traditional medicinal lore, which is always an essential source of new crude drugs for pharmacognosy and the modern industry of herbals (botanicals) and bio-based products. In this direction goes the Nagoya Protocol of 2010 as one of the major events of the past 50 years which links traditional knowledge to genetic local and typical resources. It was out of these considerations, and many more that the term “ecopharmacognosy” was proposed by Cordell in 2015 [23] and defined as “the study of sustainable, biologically active, natural resources”, in order to underline the need to bring biodiversity and knowledge to posterity. Besides many other aspects of research, working as an “ecopharmacognosist” means developing both new and established resources for nutraceuticals, cosmeceuticals as well as foods, fortified and functional foods included. Moreover, the industrial utilization of plant-based ingredients, directly handed down by popular and ethnobotanical knowledge, could contribute to developing a green bioeconomy in a global market which could have an annual trend of growth of 11%, considering also that 10–25% of prescribed drugs nowadays contain at least one active compound isolated from plants [24].

### 3.2. The Biorefinery Concept in Ecopharmacognosy: Recovery of Bioactive Compounds

In addition to the aforementioned aspects of food waste management, other factors such as global warming, scarcity of resources and the constant lookout for natural compounds and products both from industries and markets, are driving the production chains to the possible use of food wastes as a source of biomaterials. Food wastes, since containing a variety of chemical components such as polysaccharides, proteins and lipids, have thousands of significant potential applications in the market as the research has demonstrated so far. The concept of “biorefinery” defined as “the sustainable processing of biomass into a spectrum of marketable products and energy” [25] refers, analogously to the petroleum refinery, to the conversion of raw material into commercially valuable products. The production of biofuels, bioplastics, nanoparticles and bioactive compounds are some of the most relevant industrial applications with valuable economic potential. 

Plant biomass has been used for the production of biofuels for several decades, but recent developments permitted industrial organic wastes (carbohydrates from noodle wastes) as a possible source of bioethanol [26]. Another important example of biofuel production regards the conversion of cooking oil wastes into biodiesel by enzymatic transesterification [27,28].

Bioactive compounds, naturally occurring as secondary metabolites in plants, exert physiological effects in living organisms. As they are already used as nutraceuticals, cosmetics and phytotherapeutic constituents, their recovery from food waste rather than from cultivated plants represents a sustainable production alternative. In addition, recovering the industrial food waste to obtain high-added-value molecules represents nowadays a significant economic potential for the industry. For example, the conversion of citrus peel residues into high-value products would allow companies to increase their competitiveness in the market. In fact, citrus fruits, including oranges (*Citrus aurantium,* Rutaceae), lemons (*C. limon*), limes (*C. aurantiifolia*), grapefruits (*C. x paradisi*) and tangerines (*C. reticulata*) are sources of soluble sugars, cellulose and hemicellulose, pectin and D-limonene. D-Limonene, mainly used in essential oils as flavor and fragrance compound, it can be obtained after distillation of peel residues and used as a building block to generate compounds with similar structures (e.g., carveol, carvone, a-terpineol, perrillyl alcohol and perillic acid) but with possible different biological activities and, therefore, with possible different industrial applications. Pectin, one of the most important food additives used as a gelling agent and thickener, is a complex structural heteropolysaccharide found also in citrus fruits (*Citrus* spp., Rutaceae) which contain 20–30% extractable pectin. Moreover, many important flavonoids of industrial interest, including hesperidin, naringin and eriocitrin characterize the polyphenols profile of citrus peel and other solid residues of these species [12,13,14,15,16,17,18,19,20,21,22,23,24,25,26,27,28,29]. Another interesting example of industrial agri-food waste exploitation is grape pomace. In fact, after *Citrus* spp. production, *Vitis* sp. (Vitaceae), is the most produced crop in the world. Since grape pomace as food waste is approximately 20% of the weight of grapes processed, its valorization as a valuable byproduct has been the subject of numerous studies recently [30]. In fact, grape phenolics, such as flavonoids, anthocyanins, catechins, tannins and phenolic acids, are examples of extractable phytochemicals from grape pomace byproducts and they are known to have important health applications. Grape phenolics, from pomace and seeds, have been demonstrated to inhibit the oxidation of human low-density lipoproteins, neutralizing free radicals which are believed to contribute to the development of a number of health-related problems, like cardiovascular diseases and cancer [30]. Table 3 lists some more examples of industrial agri-foods residues which have been subjected to exploitation in the last 20 years. Furthermore, many more are available in literature data including valorization studies on animal-derived wastes such as fish and meat products to obtain proteins and saccharides [10]. Finally, the production of functional foods enriched in biologically active compounds is becoming increasingly popular in many countries and the potential markets are enormous.

## 4. Emerging Technologies in Ecopharmacognosy: Green Extractions

Going back to the concept of Cordell [22] regarding the meaning of ecopharmacognosy, it clearly emerges that research must necessarily consider the economic sustainability of its development toward the industrial valorization of waste/byproducts. In fact, beside its sustainable aspect, the recovery of high-value compounds must be economically profitable to be applied as an industrial production strategy. In light of all these considerations, conventional extraction processes are nowadays too expensive, time-consuming and unsustainable as they are quite laborious, requiring amounts of energy and organic solvents to be disposed of [86]. Therefore in the past years various green, safer, more efficient and often cheaper alternatives have been considered. 

Following the green chemistry approach, which appeared for the first time in 1991, six leading principles for sustainable extractions were suggested by Chemat et al. [14], summarized below.

1. Innovation through selection and use of varieties of renewable plant sources;
2. Using alternative green solvents, mainly water or agri-solvents;
3. Reducing energy consumption by energy recovery and use of innovative technologies;
4. Favoring the production of coproducts instead of wastes in the bio- and agri-refining industries;
5. Reducing producing operations favoring short, safe, robust and controlled processes;
6. Aiming for obtaining non-denatured, biodegradable and contaminants-free extracts (i.e., without heavy metals, mycotoxins, etc.).

EU environmental policy and legislation for the period 2010–2050 prioritized the use of eco-friendly solvents that need to be safe for workers, for the entire production chain process, for the environment and to be sustainably reused. To get more in-depth, in order to be considered green on the basis of the principles of green chemistry an eco-friendly solvent must meet 12 criteria [7], among which the main ones are listed below. An eco-friendly solvent must be:

- Coming from renewable feedstock;
- Recyclable with eco-efficient treatments;
- Exhibit similar solvating properties of commonly used solvents;
- Have a high boiling point and low vapor pressure;
- Be biodegradable under environmental conditions.

In 2013, Kerton and Mariotte [87] stated the following definition: “the greenest solvent, in terms of reducing wastes, is no solvent”. Following their statement, it is necessary to mention the possibility of extracting molecules without the use of solvents (solvent-free extraction). The ancient cold pressing methods used in Italy for the extraction of fixed and essential oils from Mediterranean plants are the clearest example of these techniques. Nowadays we have the possibility to use more advanced technologies, such as extractions assisted by the use of ultrasounds or microwaves, which can be a valid tool to enhance the yield of biomolecules from agri-food wastes making their extraction easier. Some of the extraction techniques, with and without the use of green solvents or adjuvant technologies, which in recent decades have proven to have the most promising advantages from both an effectiveness and sustainability point of view, are described below.

### 4.1. Ultrasound-Assisted Extraction (UAE)

Ultrasound-assisted extraction has not only been used to extract bioactive compounds (e.g., polyphenolics, anthocyanins, aromatic compounds, polysaccharides and functional compounds such), but also essential oils, steroids, and lipids from plants. Ultrasound waves (from 20 kHz to 10 MHz) have been successfully employed to extraction procedures using the cavitation effect. During the sonication process, longitudinal waves are created when a sonic wave meets a liquid medium, alternating compression and rarefaction waves. Sound waves create bubbles that grow and collapse. During the expansion cycle, these bubbles cause the expansion and diffusion of gas. During the compression cycle, the energy provided is not sufficient to retain the vapor phase in the bubble, so a rapid condensation occurs creating shock waves. These shock waves create microareas with very high temperature and pressure conditions, inducing the penetration of solvent by destroying the plant cell walls making easier the release of extractable compounds. During UAE extraction, several mechanisms of extraction have been identified and the results are the consequence of their combination: fragmentation, erosion, sonocapillarity effects, sonoporation, local shear stress and destruction of plant structures [88]. Moreover, ultrasound frequency can enhance the extraction yields with high reproducibility. Some other advantages derived by using ultrasounds as extraction strategies are low temperatures, reduction of extraction time, amount of energy and low CO_2_ emissions and, in addition, the UAE apparatus is cheaper than that of other innovative technologies. Moreover, UAE gives a crucial opportunity for a large commercial scale-up due to the availability of recently designed high-technology extraction units for large commercial operations [89].

### 4.2. Microwave-Assisted Extraction (MAE)

Microwave-assisted extraction works with electromagnetic radiations with a frequency ranging from 0.3 to 300 GHz (generally 2.45 GHz). Microwaves, due to their electromagnetic nature, generate orthogonal electric and magnetic fields. The electric field causes the heating of extracting material through dipolar rotation and ionic conduction. The former is due to the alignment on the electric field in both the solvent and the solid sample of the molecules with a dipole moment: this oscillation produces collisions among the molecules producing thermal energy. Unlike classical conductive heating methods, microwaves heat the whole sample simultaneously and this is one of the main advantages. Another advantage of microwave heating is the disruption of weak hydrogen bonds promoted by the dipole rotation of the molecules. Moreover, higher viscosity of the medium lowers this mechanism by affecting molecular rotation while the migration of dissolved ions increases solvent penetration into the matrix facilitating the solvation of the sample. The latter consists of ionic currents induced in the solution by the electric field: frictions which occur after the medium resists these currents, causing heating liberation by a Joule effect. Size and charge of the ions in the solution strongly influence this phenomenon. The effect of microwave energy is also strongly dependent on the nature of both the solvent and the solid matrix. Finally, solvents used can be polar and nonpolar but the extracting selectivity and the ability of the medium to interact with microwaves can be modulated by using them in mixtures with different polarity properties [90].

### 4.3. Pressurized Solvent Extraction (Naviglio^®^ Extractor)

Naviglio^®^ Extractor has been presented as a technological innovation in the field of solid–liquid extractions as the result of the application of a new principle called “Naviglio’s Principle” [91]. The device, proposed by D. Naviglio in 2003, is a rapid and dynamic solid–liquid extractor that applies the “Naviglio’s principle” consisting “….in using a suitable solvent, generating a negative pressure gradient followed by a rapid equilibrium condition restoring, forcing the extraction of the not chemically bound compounds contained in the solid matrix…” [91]. Naviglio^®^ Extractor can operate at room- and lower-temperature, and it works by applying a pressure increase on the surface of the liquid phase containing the solid material (matrix) to be extracted. The device consists of an extracting chamber equipped with a cylinder and a piston where, at the bottom, one porous set lets the liquid phase and soluble substances pass through, while the solid particles are blocked. The solid raw material is put in the chamber that is filled with the solvent (organic, inorganic or a mixture). During the static phase, the pressure gradient is applied to allow the system to reach equilibrium at a pressure of about 8–9 atm. When the piston is moved from its equilibrium position, the dynamic phase starts: this step is performed five times and for a brief period of time with aim of remixing the solutions and allowing the diffusion of the extracted compounds. Hence, the movement of the piston alternatively produces static and dynamic steps until the extraction process is efficiently completed. Therefore, an extraction cycle is characterized by a static and a dynamic step and, by repeating more times these operations, a complete exhausting of the solid matrix can be obtained. Finally, the main advantages of Naviglio^®^ Extractor are the use of low or room temperature that reduces the thermal stress for any heat susceptible substances present in the matrix, its ductility of use allowing an easy scale-up from laboratory research to industrial production according to demand (from bench until industrial apparatus) with large application possibilities (chemistry, medicine, agriculture, biology, etc.).

### 4.4. Supercritical Fluids Extraction (SFE)

Supercritical fluid extraction (SFE) is an extraction process based on the use of supercritical fluids as the extracting solvent from a matrix (for e.g., waste biomass). Extraction is usually from a solid matrix, but it can also be from liquid ones. Supercritical fluids are already well-established as alternative tools to extract both lipophilic and hydrophilic compounds from waste biomass. They possess liquid-like density and gas-like viscosity and these characteristics are the basis of the effectiveness of this extractive method. More than 90% of SFE has been performed using carbon dioxide (CO_2_)—since it is considered as Generally Recognized As Safe (GRAS)—as a supercritical nonpolar solvent which solvating capacity is sometimes modified by polar cosolvents such as ethanol or methanol characterized by low critical constants (extraction conditions for supercritical CO_2_ are above the critical temperature of 31 °C and critical pressure of 72 bar) [86]. Finally, the main green characteristics of this extraction process are (1) the opportunity to obtain “solvent-free” extracts and free of any solvent residue since the supercritical solvent turns into a gaseous state at room temperature; (2) the CO_2_ is nontoxic, nonflammable, odorless, tasteless, inert, and inexpensive; (3) various application possibilities in food, aromas, essential oils, cosmetics and nutraceutical industries. In fact, SFE can be used as a sample preparation step for analytical purposes or, on a larger scale, to either strip undesired material from raw material/waste biomass (for e.g., decaffeination from coffee seeds, polyphenols from exhausted coffee crude drug—*Coffea arabica, C. canephora*, Rubiaceae—, etc.) or collect fractions (for e.g., unsaponifiable fraction from oils, butters and waxes) and phytocomplexes (for e.g., terpene mixtures chemically analog to essential oils) [92].

### 4.5. Subcritical Water Extraction (SWE)

Among the more recent developments of green extraction, water at its subcritical state has been identified as an effective solvent with a number of advantages. At the temperature of 374 °C and a pressure of above 220 bar, water is considered at its supercritical state, but subcritical water extraction is performed between 100 °C and 374 °C and high pressure to keep the water liquid. By altering water conditions, this solvent changes its solvating properties which become unique and adaptable for an effective and environmentally friendly extraction. In fact, at these conditions, water decreases its polarity and becomes suitable for both polar and nonpolar compounds (this is due to a dramatic drop in the dielectric constant caused by the high temperature). As a low polar solvent, subcritical water allows us to obtain high extraction yields and reductions of extraction times. At the same time viscosity and density of water decrease too and thus enhance water penetration inside the sample matrix. Water is easily available, safe, low cost, nontoxic and noninflammable and environmentally friendly: all these advantages of use brought subcritical water extraction to receive much attention among researchers from various research fields. Furthermore, the equipment is easy to reproduce on a laboratory scale because of its technologically simple design [86,87,88,89,90,91,92,93].

### 4.6. Natural Deep Eutectic Solvents (NADES)

Firstly reported by Abbott in 2003 [94], deep eutectic solvents (DESs) are now recognized as new sustainable solvents. Because of their similarity with ionic liquids, some of their properties are the nonvolatility, the high viscosity and the nonflammability. The preparation of DESs is simple: it is sufficient to mix organic salts such as quaternary ammonium or phospohonium salt, with metal salts or hydrogen bond donors (able to create intramolecular hydrogen bonds between each other). NADESs (natural deep eutectic solvents) are DESs produced from primary metabolites commonly occurring in living cells (choline, sugars, carboxylic alcohol, etc.) which are involved in the biosynthesis and storage of various nonpolar compounds. They can be defined as green solvents because of their following properties: they have a low cost, they are simple to prepare, nontoxic, biodegradable, readily available and they can be tuned easily for specific applications. NADESs capacity to be good extractive solvents depends on their combination and physiochemical properties. Water can also be added to modify polarity. Finally, it has been estimated that 108 are the possible combinations of NADESs and their cost is comparable to one of the conventional solvents. However, some disadvantages need also to be described: they are difficult to reuse or recover; the industrial scale-up is possible only when the extract is used without purification steps, high energy consumption could be required for stirring the extraction system (solvent (s) and matrix/waste biomass) because of the high viscosity of NADESs [86,87,88,89,90,91,92,93,94,95]. New forms of NADESs have been recently described as therapeutic deep eutectic solvents (THEDESs) by Aroso et al. [96] as bioactive eutectic mixtures which contain an active pharmaceutical ingredient as one of the constituents of the mixture.

### 4.7. Enzyme-Assisted Extraction

Enzyme-assisted extraction (EAE) has gained much attention nowadays because of the need for green extraction technologies. Even if the use of enzymes to extract bioactive compounds is already well-established, its association with other technologies such as sonication and microwaves, is a promising tool. Enzymes, with their ability to disrupt cell walls, represent a good alternative to release compounds in the solution with higher yields compared to other conventional extraction methods. In particular, the use of enzymes in extraction procedures enables to reduce solvent amounts and also to extract compounds which are naturally found in a bound form with other plant structural components. Among the advantages of using enzyme-assisted extraction, there are low extraction time, high yields and low energy and low solvent consumption. Cellulases, pectinases and hemicellulases are often required to hydrolyze cell wall components and thus increase cell wall permeability. They can be derived from bacteria, fungi, animal organs or plant extracts. Appropriate operational conditions and enzymes combination are important parameters to obtain a successful result. Enzymatic extractions are subjected to continuous research: good examples are the extraction of oils, polyphenols, phenolic acids, vanillin, polysaccharides and lycopene from tomatoes (*Solanum lycopersicum,* Solanaceae) [97]. The release of bound polyphenols from plant cell walls represents a never-ending challenge among researchers that need to find green alternatives to the commonly used acid and alkaline hydrolysis conditions. Enzymes, such as feruloyl esterase, can be involved in the release of bound phenolics, in particular, phenolic acid from cereal pericarps: ferulic acid exhibited numbers of possible applications such as in health, medicine, food and cosmetic fields [98].

### 4.8. Hybrid Techniques

Beside all the innovative and sustainable extraction strategies described above, the possibility of combining two or more of them, in order to optimize results, is receiving great attention nowadays. Various combinations of extraction procedures have been investigated so far on plant materials. In particular, the combination of ultrasounds with conventional or unconventional methods has demonstrated to be effective. Ultrasound-assisted Soxhlet extraction (Sono-Soxhlet) combines the advantages of the Soxhlet extraction (availability of fresh solvent) and of ultrasounds by enhancing mass transfer and reduction of extraction time. This system has been used for the extraction of lipids from seeds, sausage products, cheese and bakery products. Another promising hybrid technique, which is fast and efficient, is made by the combination of ultrasound-assisted extraction (UAE) and microwave-assisted extraction (MAE). This combination allows us to dramatically shorten extraction time with potential great industrial applications. The mechanical ultrasonic effect promotes the release of soluble compounds from the plant matrices by disrupting cell walls while microwaves heat the sample inducing the quick migration of molecules. The simultaneous irradiation increases the penetration of the solvent into the plant matrix and can increase the solubility of compounds. This technique has been used for the extraction of pectin from pomelo peel (*Citrus maxima*, Rutaceae) [84]. Sonication has also demonstrated a positive effect when coupled with supercritical fluids (SC-CO_2_) since UAE enhances the mass transfer of ginger to the solvent used for extraction of pungent compounds increasing the yields [99]. Among many other possible combinations of technologies that can be used to extract biomolecules following the green chemistry concepts, two other strategies need to be mentioned: Ultrasound-Assisted chemical hydrolysis and Ultrasound-Assisted enzymatic hydrolysis. Since the extraction technique’s choice of the desired metabolite has to be a result of a compromise between the efficiency and reproducibility of extraction, the use of ultrasounds (alone or in combination with other techniques) has become nowadays, one of the most effective devices used to obtain green extracts because of the ease procedures and considerations of cost, time, safety and degree of automation [88].

## 5. Bound Phenolics from Waste Biomass: A Case Study

In various plant materials, phenolic compounds are commonly found in a bound form. Bound phenolics comprise, on average, 24% of the total phenolics in food, with peaks of 88% of total phenolic molecules in brown rice [100]. Acosta-Estrada [101] resumed several studies reporting food sources with nonextractable phenolics’ percentages (Table 4): these molecules are gaining attention as a considerable amount of them is often not detected due to the limitations of conventional extraction methods. The possibility of extracting bound molecules, otherwise difficult to identify, can be particularly advantageous in case of waste biomass recovery which, as a result of the industrial processing, are often apparently exhausted.

Bound phenolic acids, as well as other potential health-promoting substances, are found covalently linked to cell wall structural components such as hemicelluloses (arabinoxylans), celluloses, lignins, pectins and proteins, and often remain in the extraction’s residue which is normally discarded and unutilized. These strong bonds provide physical and chemical barriers, protection for the plant against pathogens as well as from free radicals. Ferulic acid, for example, is attached with ester linkages to the arabinoxylans of the cell wall and form ether linkages with lignin at the outermost part of the cereal caryopsis. Moreover, ferulic acid oligomers form polysaccharide-polysaccharide cross-linkages, limiting the biodegradability of the cell walls [102]. 

Agri-industrial byproducts, such as those coming from cereals and vegetables supply chains, are good sources of lignocellulosic materials rich in bound phenolics. 

As recently reviewed by Wang et al. [103], there are many possibilities for the extraction of bound phenolics, using both chemical, enzymatic and physical treatments. However, the commonly used alkaline or acid hydrolysis have demonstrated some negative aspects such as the long extraction time and partial solubilization of the molecules. By contrast, the use of mechanical extraction methods (Ultrasound-Assisted Extraction (UAE), Microwave Assisted Extraction (MAE), etc.) has demonstrated to reduce extraction times and improve the release of bound phenolic molecules.

Recently, hybrid extraction techniques have been successfully used to extract bound phenolics from plant byproducts obtaining more interesting results than those obtained with commonly used extraction techniques. Coupling sonication to alkaline hydrolysis, in fact, allows shortening extraction times enhances the phenolic yields when compared to conventional hydrolysis conditions [80,81] and enzymatic treatments [72]. Gonzales et al. [80] used Ultrasound-Assisted alkaline hydrolysis to extract bound phenolics from cauliflower (*Brassica oleracea*) wastes, one of the most consumed vegetables of the Brassicaceae family, by comparing the obtained results about extractable and nonextractable phenolic amounts. This study demonstrated that a significant amount of phenolic molecules has been underestimated before, and that cauliflower wastes have an interesting potential as a source of bioactive molecules. Ultrasonication improves the phenolic extraction by damaging the plant matrix, which, once broken, increases the surface interaction area between solvent (s) and the plant matrix to be extracted. This effect allowed the sodium hydroxide to penetrate more efficiently in the plant matrix to break ester linkages and enhance the release of the molecules. Therefore, combining sonication and alkaline hydrolysis demonstrated to be effective in the release of a higher quantity of phenolics. The effect of ultrasonication on the release of bound phenolic molecules has been also evaluated on other plant sources, such as maize germs [81]. Following the guidelines of the European Commission, maize byproducts have been investigated for their total phenolic content and antioxidant activity comparing various extraction techniques. The large amounts of *Zea mays* L. productions made in this plant an interesting and promising source of bioactives that could be valorized and reintroduced in the market as high-added-value products. Using a similar hybrid extraction method as the one previously described, maize germs exhibited a higher amount of phenolic acids and total phenolic compounds compared to the results obtained with other commonly used techniques. Moreover, by combining alkaline hydrolysis with sonication, an increase of antioxidant activity of the bound phenolic extract occurred. The results of this study confirmed the effective applicability of the previously described hydrolysis method on maize germ, which allowed to obtain enriched extracts by associating ultrasounds with alkaline hydrolysis despite the reduction of extraction times. Another interesting research, published in 2018 by Đurović et al. [103], demonstrated a positive effect of combining green extraction methods to commonly used technologies, on the release of bound phenolics from yellow soybean (*Glycine max*) seeds. In particular, combining microwave-assisted extraction and alkaline and acid hydrolysis, appeared to enhance the release of bound phenolic acids from the plant matrix as well as extraction yields and antioxidant activity. Finally, the use of enzymes could also be a good alternative to chemical hydrolysis since they are, in some cases, more effective and specific. Associating an enzymatic pretreatment with the extraction process can, therefore, help in the targeted extraction of phenolic molecules from plant sources and byproducts as demonstrated by Balasubramaniam [104]. The phenolic yield extracted from finger millet (*Eleusine coracana*), increased more than two-fold using enzymatic treatment followed by sonication compared to the commonly used method, while the yield obtained only by sonication has remained unchanged.

## 6. Conclusions: Up-Scaling and Perspectives

Green extractions, that is hybrid techniques involving the coupling of two or more extractive techniques, make it possible to improve food waste processing methods in a sustainable and more environmentally friendly way. Hybrid techniques, some of which are summarized in this review, allow industries to minimize processing and recovery times for waste materials, thus reducing working costs, energy consumption and, at the same time, increasing the extraction efficiency and quality of the products obtained. Food waste from agri-food supply chains can thus become raw materials with high-value-added and economic potential, and be reintroduced into the market through the circular economy, as suggested by the European Commission [3]. Bound phenolic molecules, previously underrated, have been attracting increasing attention in recent years because they characterize most of the waste biomass of the agricultural industry, especially cereals, and have shown great potential applications in health, nutraceutical and cosmetic fields. For these reasons, applying the most innovative and green techniques for their extraction is of particular interest nowadays. Some of the publications presented in the present review have considered the importance of scale-up of their research, proposing innovative industrial plants such as an ultrasound-microwave extractor by Đurović et al. [105]. Other studies are necessary for a careful cost-benefit analysis that will certainly be the subject of research in the short term. The lack of information on scale-up and the high investment associated with emergent extraction processes underline the initial prejudice of the industry to these new extracting technologies. However, some recent publications [106] presented rapid methods to estimate the cost of the manufacturing of extracts obtained by green extractions, especially using sonication and supercritical fluids, contributing to the possibility of increasing the application of these extractions at industrial level. Pending further research in this field, it can certainly be concluded that, given the countless risks to the environment, health and climate that the planet is experiencing in recent years, a drastic change of perspective in the field of applicative research is needed. As Cordell proposed [22,23], in fact, research has the responsibility to propose increasingly sustainable and environmentally friendly solutions for the industry to make industrial processes increasingly greener.

## Figures and Tables

**Table 1:** Five system boundaries in the food supply chain (adapted from [9]).

Vegetable Commodities and Products	
**Agricultural Production**	Losses for mechanical damage and/or spillage during harvest operations, crops separated postharvest, etc.
**Postharvest Handling and Storage**	Handling spillage and/or degradation, storage and transport during distribution
**Processing**	Spillage and degradation during processing, separated crops not suitable to process or during washing, peeling, boiling or accidental spillage
**Distribution**	Market system
**Consumption**	Household level

**Table 2:** Global food losses and food waste (adapted from [9]).

World Food Losses and Wastes (%) by Manufacturing	
Cereals	4.1
Roots and tubers	10.2
Oil crops and pulses	5.9
Fruits and vegetables	8.5
Meat	4.8
Fish	6.3
Diary	1.4

**Table 3:** Examples of target phytochemicals from fruit and vegetable wastes.

Extraction Method		Food Waste Biomass	Bioactives of Interest	References
Ultrasound-assisted extraction (UAE)		Coffee waste (*Coffea arabica; C. canephora*)	Phenolic compounds	[31]
		Pomegranate waste (*Punica granatum*)	Carotenoids	[32]
		Tomato waste (*Solanum lycopersicum*)	Pectins	[33]
		Apple pomace (*Malus domestica*)	Xyloglucan	[34]
		Grapeseed (*Vitis* spp.)	Anthocyanins, Protantocianidins	[30]
		Grape pomace	Flavonoids	[30]
		Grape leaves	Phenolic compounds	[35]
		Artichoke solid waste (*Cynara scolymus*)	Phenolic compounds	[36]
		Guava’s pulp (*Psidium guajava*)	Carotenoids	[37]
		Orange peel (*Citrus* spp.)	Phenolic compounds	[38]
Microwave-assisted extraction (MAE)		Tomato waste (*S. lycopersicum*)	Carotenoids	[39]
		Sea Buckthorn byproducts (*Hippophae rhamnoides*)	Flavonoids	[40]
		*Agaricus bisporus* L. byproducts	Erogosterol	[41]
		Sage byproducts (*Salvia officinalis*)	Phenolic compounds	[42]
		Broccoli by-products (*Brassica oleracea* var. *italica*)	Phenolics, sugars, glucosinolates	[43]
		*Citrullus lanatus* fruit rinds	Pectins	[44]
Pressurized solvent extraction (Naviglio^®^ extractor)		Tomato waste (*S. lycopersicum*)	Lycopene	[45]
		Vine-shoots (*Vitis* spp.)	Phenolic, volatile, mineral compounds	[46]
		Grape pomace (*Vitis* spp.)	Polyphenols	[30]
Supercritical fluids extraction (SFE)		Hazelnut wastes (*Corylus avellana*)	Tryglycerides	[47]
		Grape wastes (*Vitis* spp.)	Polyphenols and Fatty Acids	[48]
		Wheat bran (*Triticum aestivum*)	Phenolic compounds and tocopherol	[49]
		Sweet Potato (*Ipomoea batatas*)	Carotenoids	[50]
		Tobacco waste (*Nicotiana tabacum*)	Solanesol	[51]
Subcritical water extraction (SWE)		Rice bran (*Oryza sativa*)	Phenolic acids	[52]
		Winery byproducts (*Vitis* spp.—from wine supply chain production)	Catechins and proanthocyanidins	[53]
		Onion waste (*Allium cepa*)	Flavonoids	[54,55]
		Jackfruit peel waste (*Arctocarpus heterophyllus*)	Pectins	[56]
		Passion fruit peel *(Passiflora edulis*)	Oligosaccharides	[57]
		Grape skin (*Vitis* spp.)	Polyphenols	[58]
		Mango peel (*Mangifera indica*)	Pectins	[59]
Deep eutectic solvents (DESs and NADES)		Wine lees (*Vitis* spp.—from wine supply chain production)	Anthocyanins	[60]
		Olive pomace (*Olea europaea*)	Phenolic compounds	[61]
		Olive leaves (*O. europaea*)	Polyphenols	[62]
		Orange waste (*Citrus* spp.)	d-Limonene, Pectin and Hesperidin.	[63]
		Onion peel (*A. cepa*)	Polyphenols	[64]
Enzyme-assisted extraction		Tomato peels (*S. lycopersicum*)	Lycopene	[65]
		Raspberry wastes (*Rubus* spp.)	Phenolic compounds	[66]
		Brewer’s spent grain (*Triticum aestivum*)	Mono and dimeric ferulic acid	[67]
		Oat hulls (*Avena sativa*)	Ferulic acid	[68]
		Beer wastes	Beta-glucans	[69]
Hybrid techniques	UAE- sohxlet	Oleaginous seeds	Fixed oils	[70]
	UAE-SFE	Grape pomace (*Vitis* spp.)	Polyphenols	[71]
	UAE- enzymatic	Wheat bran (*T. aestivum*)	Arabinoxylans	[72]
		Citrus peel (*Citrus* spp.)	Pectins	[73]
		Tomato peel (*S. lycopersicum*)	Lycopene	[74]
		Olive waste (*O. europaea*)	Phenolic compounds	[75]
		Waste cooking oil		[76]
	UAE-NADESs	Olive cake (*O. europaea*)	Phenolic compounds	[77]
		Onion seed (*A. cepa*)	Phenolic compounds	[77]
		Tomato byproducts (*S. lycopersicum*)	Phenolic compounds	[77]
		Pear canning byproducts (*Pyrus communis*)	Phenolic compounds	[77]
		Tartary buckwheat hull (*Fagopyrum esculentum*)	Rutin	[78]
		Red grape pomace (*Vitis* spp.)	Phenolic compounds	[79]
		Olive leaves (*O. europaea*)	Phenolic compounds	[79]
		Wheat bran (*T. aestivum*)	Phenolic compounds	[79]
		Lemon waste peels (*Citrus limon*)	Phenolic compounds	[79]
	UAE-hydrolysis	Cauliflowers byproducts (*Brassica oleracea*)	Bound phenolics	[80]
		Maize germ (*Zea mays*)	Bound phenolics	[81]
	UAE-MAE	*Nitratia tangutorum* Bobr. byproduct	Pectins	[82]
		Walnut green husk (*Juglans regia*)	Juglone	[83]
		Pomelo peel (*Citrus maxima*)	Pectins	[84,85]

**Table 4:** Insoluble bound phenolic (data from [101]).

Bound Phenolics in Food Sources		
Source	Species	Insoluble Bound Phenolics in Total Phenolic %
Apple	*Malus domestica*	6.50
Banana	*Musa acuminate*	33.10
Cranberry pomace	*Vaccinium macrocarpon*	76.27
Orange	*Citrus sinensis*	24.30
Carrot	*Daucus carota*	37.6
Onion	*Allium cepa*	9.70
Potato	*Solanum tubersum*	39.9
Barley	*Hordeum vulgare*	70.08
Maize	*Zea mays*	85.00
Rice	*Oryza sativa*	62
Brown rice	*Oryza sativa*	88
Wheat	*Triticum* spp.	75
